# A Retrospective Multicenter Study on the Usefulness of 50 g Glucose Challenge Test in Gestational Diabetes Mellitus Screening

**DOI:** 10.31662/jmaj.2019-0072

**Published:** 2020-04-06

**Authors:** Miho Saito, Chihiro Hirai, Shintaro Makino, Jun Takeda, Shuko Nojiri, Satoru Takeda, Atsuo Itakura

**Affiliations:** 1 Department of Obstetrics and Gynecology, Juntendo University Faculty of Medicine, Tokyo, Japan; 2 Medical Technology Innovation Center Clinical Research and Trial Center, Juntendo University Faculty of Medicine, Tokyo, Japan

**Keywords:** Gestational diabetes mellitus, Glucose challenge test, Pregnancy, Random blood-glucose level

## Abstract

**Introduction:**

To clarify the usefulness of glucose challenge test (GCT), the rate of gestational diabetes mellitus (GDM) detection and perinatal outcomes were compared between the groups of random blood glucose level (RBG) and 50 g GCT in this study.

**Methods:**

The first survey was conducted at 255 institutions registered by the Kanto Society of Obstetrics and Gynecology and clinical training institutions in the Kanto Area, followed by a second survey. The included women were broadly classified into the RBG and GCT groups, according to the mid-trimester blood glucose screening method, and the perinatal outcomes of the two groups were retrospectively compared. The primary outcomes were the proportion of infants weighing 3,500 g or more and birth weight ≥90^th^-percentile infants.

**Results:**

The rate of GDM diagnosis was significantly higher in the GCT group (7.6%) than that in the RBG group (4.8%). However, no significant differences were observed in perinatal outcomes, i.e., the proportion of infants weighing 3,500 g or more or birth weight ≥90^th^ percentile.

**Conclusions:**

GCT is not superior for predicting infants weighing 3,500 g or more and birth weight ≥90^th^ percentile, as compared with RBG.

## Introduction

Gestational diabetes mellitus (GDM) is a risk factor for adverse perinatal outcomes as well as future development of type 2 diabetes mellitus in mothers. As mid-trimester blood glucose screening options for detecting GDM, the 50 g glucose challenge test (GCT) is associated with a higher detection rate than random blood glucose level (RBG) measurement ^(1), (2), (3), (4), (5)^. In 2008, the Hyperglycemia and Adverse Pregnancy Outcome study ^(6)^ reported that maternal blood glucose levels at 24–32 weeks of gestation strongly correlated with birth weight ≥90^th^ percentile, amount of body fat in infants, and C-peptide levels in the umbilical cord blood. The secondary analysis in this study revealed that the incidence rates of perinatal complications, such as preterm delivery, cesarean section, shoulder dystocia, neonatal hypoglycemia, and neonatal hyperbilirubinemia, were high in pregnant women with abnormal glucose tolerance. GDM management has been emphasized with regard to perinatal complications ^(7)^. In addition, therapeutic intervention for GDM reportedly reduces the incidence of stillbirth, neonatal death, and shoulder dystocia; number of deliveries of large-for-gestational-age infants and infants with macrosomia; and rate of emergency cesarean section compared with no intervention ^(8), (9)^. A woman delivering an infant weighing 3,500 g or more was reported to have higher risk (3.55%) of shoulder dystocia comparing with the general population (0.2%–2.1%) ^(10)^. These reports indicated the importance of an appropriate diagnosis and glycemic control. In 2009, the International Association of Diabetes and Pregnancy Study Groups (IADPSG) proposed new diagnostic criteria for GDM ^(11)^, these criteria were introduced in Japan in 2010. GCT was recommended for mid-trimester blood glucose screening. However, the selection of either RBG or GCT was left to the discretion of each institution.

Although a decrease in the incidence of perinatal complications was expected because of changes in perinatal management after the introduction of the new criteria, the incidence of macrosomia was 0.81% in 2010 and 0.78% for 2016, respectively ^(12)^. This appeared to be attributable to the inadequate detection of GDM that required intervention because the selection of screening methods was left to the discretion of institutions in Japan. Thus, we conducted this cohort study to clarify the usefulness of GCT.

## Materials and Methods

The target institutions were defined as those that belonged to the Kanto Society of Obstetrics and Gynecology and that provided labor and delivery services at perinatal care centers around Kanto area. The target study group was defined as pregnant women with singletons and GDM who received perinatal management between January and December 2015 at the institution. The first survey was conducted at 256 target institutions. A questionnaire survey was conducted by postal mail to investigate the total number of deliveries during a 1 year period in 2015, mid-trimester screening method (RBG or GCT), and possibility of participation in the second survey.

In the second survey, data from the Pregnancy Birth Registry System of the Japan Society of Obstetrics and Gynecology were collected from institutions that responded to the first survey and were able to participate in the second survey. Women with pregnancy complicated by diabetes mellitus and referral patients were excluded. According to the mid-trimester screening method, the included women were classified into two groups, the RBG and GCT groups. The rate of GDM diagnosis and perinatal outcomes were retrospectively compared.

The primary outcomes were the proportion of infants weighing 3,500 g or more and birth weight ≥90^th^ percentile in two groups. Other study variables included maternal characteristics (e.g., age, obstetric history, pre-gestational body mass index (BMI), maternal weight gain, and gestational age at delivery), proportion of pregnant women with GDM, and neonatal outcomes (e.g., birth weight, premature delivery, stillbirth, and NICU admission).

### Statistical analysis

Statistical analysis was performed using SAS version 9.4; SAS Institute, Cary, NC. Continuous variables are summarized by means and standard deviations or 95% confidence intervals (CIs). Logistic regression analysis was used to calculate the odds ratios and 95% CIs for weighing 3,500 g or more and birth weight ≥90^th^ percentile. A P value of <0.05 was considered significant. Every analysis was performed with a logistic model to correct the differences in maternal baseline characteristics such as age at delivery, non-pregnant BMI, and ratio of primiparous to parous women.

### Ethics committee approval

The study was approved by the ethics committees of Juntendo Hospital (17-010), and written consent has been obtained from all patients.

## Results

**Figure 1.**
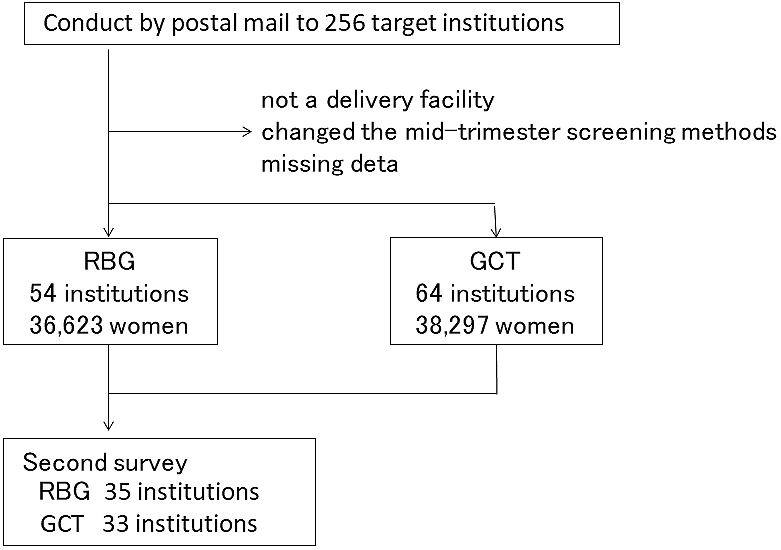
Enrollment and assignment of this study in the first survey.

In the first survey, responses were obtained from 138 of 256 institutions (53.9%). Of the 138 institutions, 122 provided labor and delivery services. The total number of deliveries was 79,668. RBG and GCT were performed in 36,623 and 38,297 women at 54 and 64 institutions after institutions that had changed their mid-trimester screening methods in the past or provided no response were excluded. The number of pregnant women undergoing either method was nearly the same (Figure 1).

**Figure 2.**
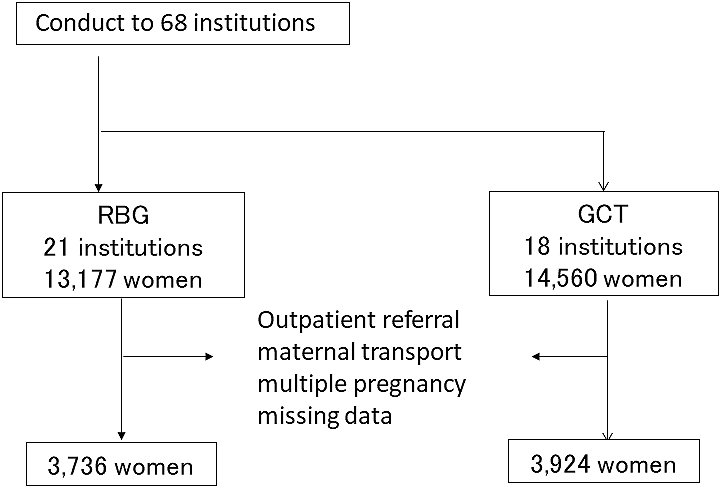
Enrollment and assignment of this study in the second survey.

A request for participation in the second survey was sent to 68 institutions, responses were obtained from 42 institutions (61.8%). Finally, valid responses were provided from 39 institutions (21 and 18 institutions performing RBG and GCT: 13,177 and 14,560 women). The type of participating institutions according to the screening methods was as follows: 1) the institutions performing RBG included four general perinatal care centers, 10 regional perinatal care centers, and seven others, in those GCT performed at five, seven, and six institutions, respectively. From the patients described in the survey responses, women referred to the outpatient clinic and those transferred to the target institutions were excluded, whereas only those who had been managed pregnancy from the early stage at the target institutions were included. In addition, we excluded women who had preterm delivery before 24 weeks of gestation and did not undergo mid-trimester screening, those with multiple pregnancies, and those with missing data. A total of 3,736 and 3,924 women in the RBG and GCT groups, respectively, were compared (Figure 2).

### Maternal characteristics

The mean age at delivery was 32.7 ± 5.1 and 33.4 ± 4.9 years in the RBG and RCT groups, respectively. The GCT group comprised older women, with lower BMI, and more primiparous women than the RBG group. GDM was diagnosed in 175 women (4.8%) in the RBG group and 298 women (7.6%) in the GCT group. The proportion of pregnant women with GCT was significantly higher in the GCT group than in the RBG group (Table 1).

**Table 1.** Characteristics of Cases and Rate of GDM Diagnosis.

	RBG (n = 3736)	GCT (n = 3924)	P value
Age（years）	32.7 ± 5.1	33.4 ± 4.9	<0.001
Primipara/Multipara（n）	1726/2010	2160/1764	<0.001
Pregestational BMI (kg/m^2^)	21.2 ± 3.3	20.7 ± 3.1	<0.001
Gestational age at delivery（weeks）	38.5 ± 1.7 (24–42)	38.6 ± 1.6 (24–42)	n.s.
The rate of GDM diagnosis (%)	4.8	7.6	<0.05

Data are presented as number (%), mean ± standard deviation (range). *, Random blood glucose group; §, 50 g glucose challenge test group

### Perinatal outcomes

The incidence of macrosomia was 0.8% in the RBG group and 1.1% in the GCT group. No significant differences were observed in the proportions of infants weighing 3,500 g or more and birth weight ≥90^th^ percentile. The proportions of infants with an Apgar score of 7 or lower were 6.3% and 5.4%, respectively, at 1 min and 1.8% and 1.5%, respectively, at 5 min. There were no significant differences in the proportions of premature delivery, stillbirth, and NICU admission (Table 2).

**Table 2.** Perinatal Outcomes.

	RBG (n = 3736)	GCT (n = 3924)	P value
>3,500 g n (%)	383 (10.3%)	371 (9.5%)	n.s.
Birth weight >90th percentile n (%)	403 (10.7%)	402 (10.2%)	n.s.
Macrosomia n (%)	32 (0.8%)	41 (1.1%)	n.s.
Premature delivery n (%)	233 (6.2%)	253 (6.5%)	n.s.
Stillborn n (%)	12 (0.32%)	8 (0.20%)	n.s.
NICU admission^§^	362 (9.7%)	331 (8.4%)	n.s.

Data are presented as number (%). RBG, random blood glucose group; CTG, 50 g glucose challenge test group

Analyses were performed with a logistic model to correct the differences in maternal baseline characteristics, such as age at delivery, non-pregnant BMI, and ratio of primiparous to parous women. The main outcomes were the proportion of infants weighing 3,500 g or more, birth weight ≥90^th^ percentile. Although a high BMI was identified as a risk factor for delivering an infant weighing 3,500 g or more or birth weight ≥90^th^ percentile, the difference in the screening methods was not a risk factor (Table 3 and 4).

**Table 3.** Logistic Model for Birth Weight >90th Percentile.

	Univariate	95%CI	Multivariate	95%CI
Age	1.005	0.990–1.020	1.005	0.989–1.021
Pre-gestational BMI	1.094	1.072–1.116	1.096	1.074–1.119
Multipara (ref: primipara)	1.058	0.914–1.224	1.164	0.992–1.365
Screening (ref: RBG)	1.056	0.913–1.222	1.010	0.863–1.182

RBG, random blood glucose group

**Table 4.** Logistic Model for >3,500 g.

	Univariate	95% CI	Multivariate	95% CI
Age	1.008	0.993–1.023	0.999	0.983–1.015
Pre-gestational BMI	1.093	1.071–1.115	1.097	1.068–1.112
Multipara (ref: primipara)	0.789	0.678–0.918	1.697	0.728–1.010
Screening (ref: RBG)	1.094	0.941–1.271	1.006	0.878–1.212

RBG, random blood glucose group

## Discussion

This the first multicenter study is to examine whether the use of different methods of mid-trimester blood glucose screening can improve perinatal outcomes for Japanese women. GCT is an internationally recommended mid-trimester screening method in terms of identification of mothers at risk of developing diabetes mellitus and the cost of GDM diagnosis ^(13)^. However, the present study revealed that GCT is not performed at approximately half of the institutions. Because the target institutions were randomly selected, the proportion of hospital-level facilities performing GCT can be assumed to be comparable with the corresponding proportion in the present study. Although the rate of GDM diagnosis was high in the GCT group as observed, the present cohort showed no significant differences in the perinatal outcomes, i.e., the proportion of infants weighing 3,500 g or more and birth weight ≥90^th^ percentile.

Studies conducted in Australia ^(8)^ and the United States ^(9)^ reported that screening for GDM with GCT and treating identified women with GDM improved perinatal outcomes. In the Australian study, the mean BMI was 26.8 kg/m^2^ in the treated women and 26.0 kg/m^2^ in the untreated women, with the American study reporting 30.1 and 30.2 kg/m^2^, respectively. These values were higher than the mean BMI observed in the present study. Furthermore, Catalano et al. ^(14)^ reported that a combination of obesity and GDM elevated the risk of increased birth weight. However, because the proportions of pregnant women with a BMI of ≥25 kg/m^2^ in the present study were as low as 9.4% and 7.3% in the RBG and GCT groups, respectively, the effect of diagnosis and treatment of GDM on perinatal complications appeared to be low in this population.

The prevalence rates of GDM and glucose tolerance are reported to vary among ethnic groups ^(15), (16)^. A report from Japan that the treatment of GDM in only obese pregnant women could reduce birth weight ≥90^th^ percentile ^(17)^. The effect of medical interventions may exhibit for Japanese people insufficiently.

In recent years, the risk of postpartum development of diabetes mellitus has become to be highly recognized, even in women with GDM diagnosis based on the new criteria (IADPSG criteria) ^(18)^. This meta-analysis shows that GDM women, based on any diagnostic strategy as well as among Asian, have postpartum risk of type 2 diabetes. Thus, GCT with high diagnostic rate for GDM among this cohort would be beneficial for mothers.

In addition, we examined whether any biases were involved in the differences in the baseline characteristics. Regarding selection bias, because the study included institutions registered by the Kanto Society of Obstetrics and Gynecology and teaching institutions providing initial clinical training, only medium- to large-sized institutions participated. However, there was no selection bias because the participating institutions were randomly selected from institutions of similar sizes responding that they could participate. Second, the present cohort study may be biased in its selection of screening methods. However, because they were selected at the discretion of each institution, maternal baseline characteristics, namely, preconceptional BMI, age, and the ratio of primiparous to parous women, which are presumably associated with birth weight of infants (a primary outcome), are not confounders for the selection of screening methods. Although significant differences were observed in these variables between the two groups, the differences in numerical values were small. We therefore considered that there was no difference in the baseline characteristics, and the large sample size contributed to the low P values. Because objective measurements were compared in this cohort study, there was little influence of information bias.

The study has some limitations. Because only data from the perinatal database were analyzed, the conditions of women at the time of GDM diagnosis (e.g., gestational age at the time of diagnosis and results of oral glucose tolerance tests) and details of the treatment of GDM (e.g., the rate of insulin use and insulin dose) were not examined. In addition, we were unable to analyze perinatal complications, such as preeclampsia, polyhydramnios, shoulder dystocia, and neonatal hypoglycemia. To investigate the usefulness of GCT for predicting perinatal outcomes, it will be necessary to collect and compare more detailed data, including data on complications that were not examined in the present study.

In conclusion, compared with RBG, GCT is not superior in predicting the infant weighing 3,500 g or more or birth weight ≥90^th^ percentile.

## Article Information

### Conflicts of Interest

None

### Sources of Funding

This work was supported by the Kanto-Rengo Society of Obstetrics and Gynecology.

### Acknowledgement

We thank the following institutions for participating in this study:

Aiiku Hospital, Chiba University, Fuji City General Hospital, Funabashi Central Hospital, Gunma University Graduate School of Medicine, Hokushin General Hospital, JCHO Tokyo Yamate Medical Center, JR Tokyo General Hospital, Juntendo University Shizuoka Hospital, Juntendo University School of Medicine, Juntendo University Urayasu Hospital, Kanto Rosai Hospital, Keiyu Hospital, Kimitsu Chuo Hospital, Kobari General Hospital, Kohsei Chuo General Hospital, National Defense Medical College, National Hospital Organization Chiba Medical Center, National Hospital Organization Yokohama Medical Center, NTT Medical Center Tokyo, Saiseikai Kawaguchi General Hospital, Saiseikai Yokohamashi Nanbu Hospital, Saitama City Hospital, Saitama Medical University Hospital, Sanikukai Hospital, Shizuoka Children's Hospital, St. Marianna University School of Medicine Hospital, Suwa Red Cross Hospital, Tachikawa Hospital, Tachikawa Sogo Hospital, Toho University Omori Medical Center, Tokai University School of Medicine, Tokyo Medical and Dental University, Tokyo Metropolitan Bokutoh Hospital, Tokyo Metropolitan Health and Medical Corporation Toshima Hospital, Tokyo Metropolitan Ohtsuka Hospital, Tokyo Metropolitan Tama Medical Center, Yokohama City University Hospital, and Yokohama Rosai Hospital

### Author Contributions

MS was the principal investigator and drafted the manuscript with the help of CH and SM. CH supervised the survey and contributed to the data collection. SM and SN contributed to developing the sampling strategy, analyzing data, and revising the manuscript. JT contributed to the interpretation of the data and revising the manuscript. ST contributed to the acquisition of the data. MS contributed to gathering local information and acquisition of the data. SM and AI contributed to the conception of the study and interpretation of the data. All authors read and approved the final manuscript.

### Approval by Institutional Review Board (IRB)

17-010 (Juntendo Hospital)
